# Associations of soil bacterial diversity and function with plant diversity in *Carex* tussock wetland

**DOI:** 10.3389/fmicb.2023.1142052

**Published:** 2023-03-01

**Authors:** Yan Li, Chuanqi Shi, Dan Wei, Junnan Ding, Nan Xu, Liang Jin, Lei Wang

**Affiliations:** ^1^Institute of Plant Nutrition, Resources and Environment, Beijing Academy of Agriculture and Forestry Sciences, Beijing, China; ^2^Heilongjiang Province Key Laboratory of Cold Region Wetland Ecology and Environment Research, Harbin University, Harbin, Heilongjiang, China

**Keywords:** soil bacteria, bacterial diversity, bacterial function, plant diversity, tussock wetland

## Abstract

Some species of *Carex* can form tussocks, which are usually distributed in valleys and flood plains. The soil microbial community diversity and function of micro–habitats formed by tussocks are associated with plant diversity, and research on these associations can guide *Carex* tussock wetland restoration. In this study, we selected tussock wetlands dominated by *Carex appendiculata*, including natural wetlands (*NW*), artificially restored wetlands (*ARW*), and naturally restored wetlands (*NRW*), and investigated plant diversity. Soil samples were collected from the quadrats of each sample plot with the maximum (*ma*), median (*me*), and minimum (*mi*) plant Shannon index values, and high-throughput sequencing was used to analyze the bacterial community composition, diversity, and functions. The plant diversity indexes of neither *ARW* nor *NRW* significantly differed from that of *NW*, but the companion species in *NRW* were hygrophytes and mesophytes, in contrast to only hygrophytes serving as companion species in *NW* and *ARW*. The soil bacterial communities at the operational taxonomic unit level of the nine quadrats with different plant Shannon index values significantly (*p* < 0.01) differed. The relative abundances of the dominant phyla (Proteobacteria, Chloroflexi, and Bacteroidetes) and the dominant genera (*Geobacter*, *Sideroxydans*, and *Clostridium* except for unassigned genera) significantly (*p* < 0.05) differed under the different levels of plant diversity. The plant Shannon index, soil moisture content, total organic carbon, N, and P were significantly (*p* < 0.05 or *p* < 0.01) correlated with the bacterial Shannon index. The phylogenetic diversity of the bacterial community in *NW* was significantly (*p* < 0.0001) different from those in *ARW* and *NRW*, and that in *ARW* was also significantly (*p* < 0.05) different from that in *NRW*. The functional groups of bacterial communities associated with plant diversity. In the *NWme*, *ARWme*, and *NRWme* bacterial communities, the relative proportions of functional groups related to soil N cycle were higher, but those related to soil S and C cycles were lower. Considering the rehabilitation of both plant and microbial communities, the methods used for establishing the *ARW* are recommended for *Carex* tussock wetland restoration.

## Introduction

Wetlands are areas with permanent or seasonal water bodies dominated by emergent vegetation and/or with permanently water-logged soil (Janse et al., 2019). They play important roles in storing C and N, protecting species diversity, and ensuring regional ecological security (Hefting et al., 2013). At the global scale in particular, tussocks, defined as individuals of graminoid species growing in clumps, tufts, hummocks, or bunches, are often distributed within inland freshwater marshes, river wetlands, and plateau wetlands in the north temperate zone (Levine, 2000; Crain and Bertness, 2005). For example, in the alternately wet–dry regions of wetlands in Northeast China, individuals of *Carex appendiculata*, *C. meyeriana*, and *C. schmidtii* can form tussocks, or dense hummocks; after their roots grow and rot repeatedly, tiller nodes are elevated, and long–term condensates form peat (Wang et al., 2018; Zhang et al., 2020; Qi et al., 2021). A tussock is usually cylindrical, with an average height of 25.3 cm, sometimes up to 1 m. The diameter of a tussock is 30–40 cm, and their density is generally 2–22 tussocks per 9 m^2^ (Tsuyuzaki and Tsujii, 1992; Zhang et al., 2020). Tussock species are often associated with *Deyeuxia purpurea*, *Glyceria spiculosa*, and *Polygonum hydropiper*, among others, forming tussock wetland vegetation (Wang M. et al., 2021). Owing to the morphological characteristics of tussocks, tussock wetlands can form unique surface micro–landforms, which can effectively increase the wetland surface area and environmental heterogeneity, provide sufficient growth space for wetland plants, further improve plant photosynthesis efficiency, and maintain high plant diversity (Peach and Zedler, 2006; Qi et al., 2019; Wang M. et al., 2021).

As important components of wetland ecosystems, microorganisms are crucial to substance transformation and energy flow, which promote the differentiation and succession of wetland ecosystems. Changes in wetland environment will cause changes in soil microbial communities (Xu et al., 2004; Lyons and Lindo, 2020). Especially in the micro-habitat formed by tussocks, the different hydrothermal conditions present affect the composition of the soil microbial community, and the soil microbial community thus exhibits heterogeneity. The number of bacteria in the topsoil of tussock wetland will gradually increase with decreases in water content and increases in temperature (Xu et al., 2004; Liu et al., 2018). Plant diversity and composition are associated with soil microbial community (Zak et al., 2003; Shi et al., 2021). In the rehabilitated wetland, vegetation type can effect on soil microbial dynamics and carbon emissions (Bonetti et al., 2021). Low wetland plant diversity and litter loss lead to the reductions of nutrients available to microorganisms, the number of microorganisms and microbial diversity (Chen et al., 2021). Therefore, revealing plant–microorganism association is fundamental to studying wetland functions, protection and restoration.

The formation of tussock wetland is the result of long-term comprehensive plant–microorganism–soil environment interaction (Mark et al., 1985; Peach and Zedler, 2006). Once damaged, it is difficult to restore tussock wetland in a short time (Qi et al., 2021). At present, researchers have evaluated a variety of restoration methods. However, which method is more suitable requires in-depth study of this comprehensive interaction. In particular, a specific assessment of the soil microbial community of *Carex* tussock wetlands is still insufficient. Therefore, the present study had the following aims: (1) to reveal the characteristics of plant diversity and soil bacterial community diversity of *Carex* tussock wetland, (2) to analyze the associations of soil bacterial diversity and function with plant diversity, (3) to provide suggestions for the restoration of *Carex* tussock wetland based on both plant and soil bacterial community rehabilitation.

## Materials and methods

### Study sites description

The study sites were in natural wetland (*NW*), artificially restored wetland (*ARW*), and naturally restored wetland (*NRW*), habitats located in Jinhewan Wetland Botanical Garden, Taiyangdao Park, and Alejin Wetland Park, respectively, in Harbin, Northeastern China. It has a semi–humid continental monsoon climate in the middle temperate zone. The dominant species of the three tussock wetlands was *C. appendiculata*. The *ARW* establishment began in 2008. After *in situ* longitudinal cutting of native tussock, root cloning and transplanting were conducted to expand the area of tussock wetlands (Qi et al., 2019, 2021). Naturally restored wetland establishment started from the returning farmland to wetland project in 2008, and the vegetation was naturally restored on the basis of the original tussock wetland. The water levels of *ARW* and *NRW* sites were controlled to avoid seasonal flooding. The three sample plots have peat soil.

### Plant community diversity determination and soil sampling

In June, 2018, the fruiting period of *C. appendiculata*, the quadrat method (with 3 × 3 m quadrats) was used to investigate plant diversity. Nine quadrats were set up along a line transect for every sample plot. The minimum distance between any two quadrats was 10 m. Based on the plant diversity records, the three quadrats with the maximum (*ma*), median (*me*), and minimum (*mi*) plant Shannon index value in each sample plot were selected, respectively, to represent the plant diversity characteristics of each sample plot, and 500 g samples of the 10–20 cm layer soil were obtained from each quadrat using the five-point (i.e., apexes and center) method. Additionally, the *mi* of the each sample plot was located in an area with 5–15 cm of surface ponding.

### Soil physicochemical indexes determination

In the laboratory, moisture content (MC) was determined using the drying method (at 105°C); soil bulk density (BD) was determined using a cutting ring (volume, 100 cm^3^); pH value was determined [water:soil, 2.5:1 (*v*:*w*)] using the composite electrode method (INESA PHS-3C, Shanghai, China); total organic C (TOC) content was determined using a TOC analyzer (Multi N/C 2100; Analytik Jena, Jena, Germany); total N (TN) content was determined by the semimicro Kjeldahl method; alkali-hydrolyzed N (AN) content was determined by the alkaline diffusion method; total P (TP) content was determined by the molybdenum antimony colorimetric method; available P (AP) content was determined by the sodium bicarbonate extraction-molybdenum antimony anticolorimetric method; total K (TK) content was determined by the flame spectrophotometry method; available K (AK) content was determined by the ammonium acetate extraction-flame photometric method (Shi et al., 2021). All index measurements were repeated three times.

### Soil sample DNA extraction

From 0.5 g soil samples, the total DNA was extracted using a PowerSoil^®^ DNA Isolation Kit (MO BIO Laboratories, Inc. Carlsbad, CA, United States) following the manufacturer’s instructions. A NanoDrop^™^ 2000 UV–Vis spectrophotometer (Thermo Scientific, Wilmington, DE, United States) was used to detect the DNA concentration and purity. Then 1% agarose gel electrophoresis was used for DNA quality testing.

### Polymerase chain reaction amplification and sequencing

Polymerase chain reaction amplification was performed using bacterial 16S rRNA V4–V5 primers (515F, 5′-GTGCCAGCMGCCGCGGTAA-3′, 926R: 5′-CCGTCAATTCMTTTGAGTTT-3′). In the first step, the volume of the PCR system was 50 μl, containing 10 μl of 5 × Buffer, 1 μl of dNTP (10 mmol L^−1^), 1 U of Phusion^®^ High–Fidelity DNA Polymerase, 0.4 μl of bovine serum albumin (BSA; 10 mg mL^−1^), 1 μl of each F and R primer (10 μmol L^−1^), 25 ng of template DNA, and sufficient ddH_2_O to complete the 50 μl volume. The PCR thermocycling protocol was as follows: 94°C denaturation for 2 min; 25 cycles of 94°C for 30 s, 56°C annealing for 30 s, and 72°C elongation for 30 s; 72°C final extension for 5 min. The PCR products were detected by 2% agarose gel electrophoresis and recovered using a DNA gel recovery kit (Axygen Biosciences, Union City, CA, United States). In the second step, the volume of the PCR system was 40 μl, containing 8 μl of 5 × Buffer, 1 μl of dNTP (10 mmol L^−1^), 0.8 U Phusion^®^ High–Fidelity DNA Polymerase, 0.4 μl of BSA (10 mg mL^−1^), 1 μl of each F and R primer (10 μmol L^−1^), 5 μl of PCR products of the first step (for DNA templates), and sufficient ddH_2_O to complete the 40 μl volume. The PCR parameters were the same as the first step, though with 8 cycles instead of 25. Finally, the PCR products were also detected and recovered as described for the first step and then quantified using the Quanti Fluor^™^-ST Blue Fluorescence Quantification System (Promega, Madison, WI, United States). The samples were sent to Genesky Biotechnologies Inc. (Shanghai, China) for sequencing using the NGS Illumina MiSeq high–throughput 2 × 300 bp sequencing platform (Illumina, San Diego, CA, United States). Each sample was analyzed in triplicate.

### Statistical analysis

MiSeq sequencing generated paired–end reads, and optimized data were obtained using the method of Liu et al. (2019). All data were submitted to the NCBI Sequence Read Archive database (Accession number: PRJNA921719). Following Edgar (2013), operational taxonomic unit (OTU) clustering of nonrepetitive sequences was performed at a 97% similarity threshold using the UPARSE pipeline. The taxonomic analysis of OTUs was performed by applying the Ribosomal Database Project Classifier (Wang et al., 2007) and the Bayes algorithm with a 0.7 confidence level, and the taxonomic identification database was the SILVA 138/16 s bacteria database (Quast et al., 2013). The relative abundance of each OTU was determined.

According to the quadrats records, plant diversity indexes, including the Shannon index, Pielou index and Simpson’s index, were calculated following Palaghianu (2016). The Chao1 index, Shannon index, and Simpson’s index of the bacterial community diversity of each sample were analyzed using Mothur v1.39.5 software (Schloss, 2020). Null model analysis was conducted using the “picante” package in R v4.2.1 (Kembel et al., 2010) to classify the relative importance of stochastic processes and deterministic processes in the phylogenetic diversity of bacterial communities. The β–nearest taxon index (βNTI) score was used to measure the variation in the relative importance of these processes, with|βNTI| < 2 indicating stochastic processes were dominant, and |βNTI| > 2 indicating deterministic processes were dominant (Liao et al., 2022), and Bray–Curtis dissimilarity was used to measure significant differences among the three sample plots. Using the “vegan” package (2.6–4) in R v4.2.1, nonmetric multidimensional scaling (NMDS) analysis was performed based on Bray–Curtis distance at the OTU level, and the significance of differences was assessed by analysis of similarities (ANOSIM) with 999 permutations. Analysis of variance (ANOVA) was performed using SPSS version 17.0 software (SPSS Inc. Chicago, IL, United States) to analyze the significance of differences in diversity indexes, soil physicochemical indexes, and the relative abundance of taxa among different treatments. Additionally, Pearson correlation analysis was performed to analyze the correlation between the diversity index and soil physicochemical index. Bar plots were drawn using Office Excel 2016 (Microsoft Corp., Redmond, WA, United States) based on the relative abundance of dominant taxa. Linear discriminant analysis of effect size (LEfSe) was performed to detect taxa with significant differences in relative abundance among treatments using the nonparametric factorial Kruskal–Wallis sum-rank test, and linear discriminant analysis (LDA) was used to estimate the size of the effect of each taxon on the difference in relative abundance.^1^ Soil bacterial functions were predicted using FAPROTAX (Louca et al., 2016).

## Results

### Plant community diversity

In each of the three sample plots, the dominant species of the plant community was *C. appendiculata*. Its coverage was above 70% in every quadrat. The companion species of all the quadrats spanned 24 families, 56 genera, and 81 species of herbaceous plants. Cyperaceae (14 species), Poaceae (10 species) and Asteraceae (9 species) were the dominant families among companion species, and the species of these families, for example, *Cyperus orthostachyus*, *Echinochloa crus-galli*, and *Bidens maximowicziana*, were highly abundant. Most of the companion species in *NW* and *ARW* quadrats were hygrophytes, belonging to Cyperaceae and Poaceae, while the companion species in *NRW* quadrats were hygrophytes and mesophytes, belonging to Cyperaceae, Poaceae and Asteraceae.

Based on the ANOVA results (Table 1), the differences in the plant diversity indexes, including the Shannon index, Pielou index, and Simpson’s index, among the three sample plots were not significant (*p* > 0.05). Specifically, there was no significant difference in plant diversity between *NW* and either *ARW* or *NRW*. The plant diversity index values in Table 1 show the Pielou and Shannon indexes of plants had the same trend, contrasting with the trend in Simpson’s index.

**Table 1:** Plant community diversity indexes.

Index	*NW*			*ARW*			*NRW*		
	*ma*	*me*	*mi*	*ma*	*me*	*mi*	*ma*	*me*	*mi*
Shannon	0.980 ± 0.155 a			0.891 ± 0.105 a			0.951 ± 0.104 a		
	1.166	0.975	0.757	1.061	0.899	0.722	1.056	0.962	0.753
Pielou	0.708 ± 0.109 a			0.628 ± 0.080 a			0.681 ± 0.076 a		
	0.841	0.606	0.546	0.765	0.649	0.521	0.762	0.598	0.543
Simpson	0.460 ± 0.086 a			0.484 ± 0.046 a			0.447 ± 0.070 a		
	0.346	0.530	0.599	0.429	0.470	0.586	0.374	0.454	0.613

Data represent the means ± standard deviations. Analysis of variance (with Duncan’s multiple comparison test) was used to test the significance of differences. Values labeled with the same lowercase letter were not significantly different (*p* > 0.05). *NW*, natural wetland; *ARW*, artificially restored wetland; *NRW*, naturally restored wetland; *ma*, maximum plant Shannon index quadrat; *me*, median plant Shannon index quadrat; *mi*, minimum plant Shannon index quadrat.

### Soil physicochemical properties

Soil physicochemical indexes are shown in Table 2. Among the three sample plots, the differences in MC were not significant (*p* > 0.05), except for *NWme* being significantly (*p* < 0.05) lower than *ARWme*. However, among the different quadrats of each sample plot, the MC of *mi* was significantly higher than those of *ma* and *me*. The BD of *NW* was higher than those of *ARW* and *NRW*, although the differences among *mi* were not significant. In each sample plot, the BD values of *ma* and *me* were significantly higher than those of *mi*, because the surface ponding in *mi* led to an increase in MC but a decrease in BD.

**Table 2:** Physicochemical indexes of soil.

Index	Quadrat	Sample plot								
		*NW*			*ARW*			*NRW*		
MC %	*ma*	a	25.60 ± 1.84	b	a	26.52 ± 1.73	b	a	23.42 ± 2.75	b
	*me*	b	25.68 ± 2.64	b	a	28.32 ± 1.50	b	ab	26.94 ± 0.73	b
	*mi*	a	55.95 ± 5.61	a	a	60.10 ± 5.13	a	a	61.67 ± 3.99	a
BD (g cm^−3^)	*ma*	a	1.15 ± 0.06	a	b	1.01 ± 0.10	a	b	0.94 ± 0.05	a
	*me*	a	1.18 ± 0.09	a	b	1.02 ± 0.11	a	b	1.00 ± 0.11	a
	*mi*	a	0.67 ± 0.07	b	a	0.59 ± 0.05	b	a	0.60 ± 0.05	b
pH	*ma*	a	6.50 ± 0.10	b	a	6.44 ± 011	b	a	6.46 ± 0.12	b
	*me*	a	6.54 ± 0.08	b	b	6.37 ± 0.06	b	ab	6.47 ± 0.13	b
	*mi*	a	6.73 ± 0.15	a	a	6.67 ± 0.03	a	a	6.75 ± 0.12	a
TOC (g kg^−1^)	*ma*	a	53.16 ± 2.78	a	a	50.50 ± 1.82	a	a	52.72 ± 3.08	a
	*me*	a	54.10 ± 2.58	a	a	49.15 ± 2.28	a	a	50.46 ± 5.30	a
	*mi*	a	43.79 ± 1.59	b	a	43.36 ± 1.68	b	a	45.06 ± 1.63	b
TN (g kg^−1^)	*ma*	a	3.59 ± 0.05	a	a	3.52 ± 0.07	a	a	3.50 ± 0.15	a
	*me*	a	3.53 ± 0.06	a	a	3.42 ± 0.12	a	a	3.37 ± 0.22	ab
	*mi*	a	3.17 ± 0.08	b	a	3.24 ± 0.16	b	a	3.21 ± 0.10	b
AN (mg kg^−1^)	*ma*	a	321.25 ± 33.31	a	b	269.01 ± 22.34	a	b	271.26 ± 15.76	a
	*me*	a	209.05 ± 21.04	b	a	241.24 ± 34.35	a	a	228.98 ± 32.99	b
	*mi*	a	198.93 ± 20.58	b	a	194.16 ± 15.45	b	a	222.47 ± 23.52	b
TP (g kg^−1^)	*ma*	a	0.84 ± 0.77	a	a	0.88 ± 0.10	a	a	0.83 ± 0.03	a
	*me*	a	0.77 ± 0.07	ab	a	0.80 ± 0.13	ab	a	0.84 ± 0.06	a
	*mi*	a	0.72 ± 0.04	a	a	0.70 ± 0.05	b	a	0.68 ± 0.14	b
AP (mg kg^−1^)	*ma*	a	82.13 ± 5.25	a	a	77.30 ± 9.38	a	a	74.78 ± 10.19	ab
	*me*	ab	79.99 ± 7.39	a	b	69.38 ± 7.66	ab	a	83.87 ± 9.82	a
	*mi*	a	69.32 ± 5.11	b	a	65.00 ± 2.99	b	a	70.18 ± 7.36	b
TK (g kg^−1^)	*ma*	b	1.01 ± 0.15	ab	a	1.35 ± 0.09	a	a	1.34 ± 0.18	a
	*me*	a	1.14 ± 0.30	a	a	1.25 ± 0.26	ab	a	1.31 ± 0.22	ab
	*mi*	a	0.83 ± 0.13	b	a	1.03 ± 0.25	b	a	1.09 ± 0.11	b
AK (mg kg^−1^)	*ma*	a	309.19 ± 22.35	a	a	297.09 ± 29.31	ab	a	292.97 ± 40.81	a
	*me*	a	290.66 ± 17.47	a	a	319.65 ± 26.08	a	a	323.71 ± 29.83	a
	*mi*	a	254.65 ± 19.28	b	a	271.47 ± 20.46	b	a	276.82 ± 32.76	a

Data represent the means ± standard deviations. Analysis of variance (with Duncan’s multiple comparison test) was used to test the significance of differences. Values labeled with different lowercase letters were significant different (*p* < 0.05). The left letters correspond to comparisons of the differences among the three sample plots, and the right letters correspond to comparisons of the differences among the three quadrats within each sample plot. MC, moisture content; BD, bulk density; TOC, total organic C; TN, total N; AN, alkali–hydrolyzed N; TP, total P; AP, available P; TK, total K; AK, available K. *NW*, natural wetland; *ARW*, artificially restored wetland; *NRW*, naturally restored wetland; *ma*, maximum plant Shannon index quadrat; *me*, median plant Shannon index quadrat; *mi*, minimum plant Shannon index quadrat.

The soil was weakly acidic to neutral. The pH value of *NWme* was significantly higher than that of *ARWme*, and pH values of *mi* were significantly higher than those of *ma* and *me*, i.e., the soil pH value was closer to neutral in the quadrats with surface ponding.

The AN of *NWma* was significantly higher than those of *ARWma* and *NRWma*, but the TK of *NWma* significantly lower. The AP of *ARWme* was significantly lower than that of *NRWme*. The TOC, TP, and AK of the three sample plots did not significantly differ, respectively. Among the different quadrats of each sample plot, the nutrient contents of *mi* were lower than those of *ma* and *me*, namely, the lower the plant diversity, the lower the nutrient content.

### Soil bacterial community structure

**Figure 1:**
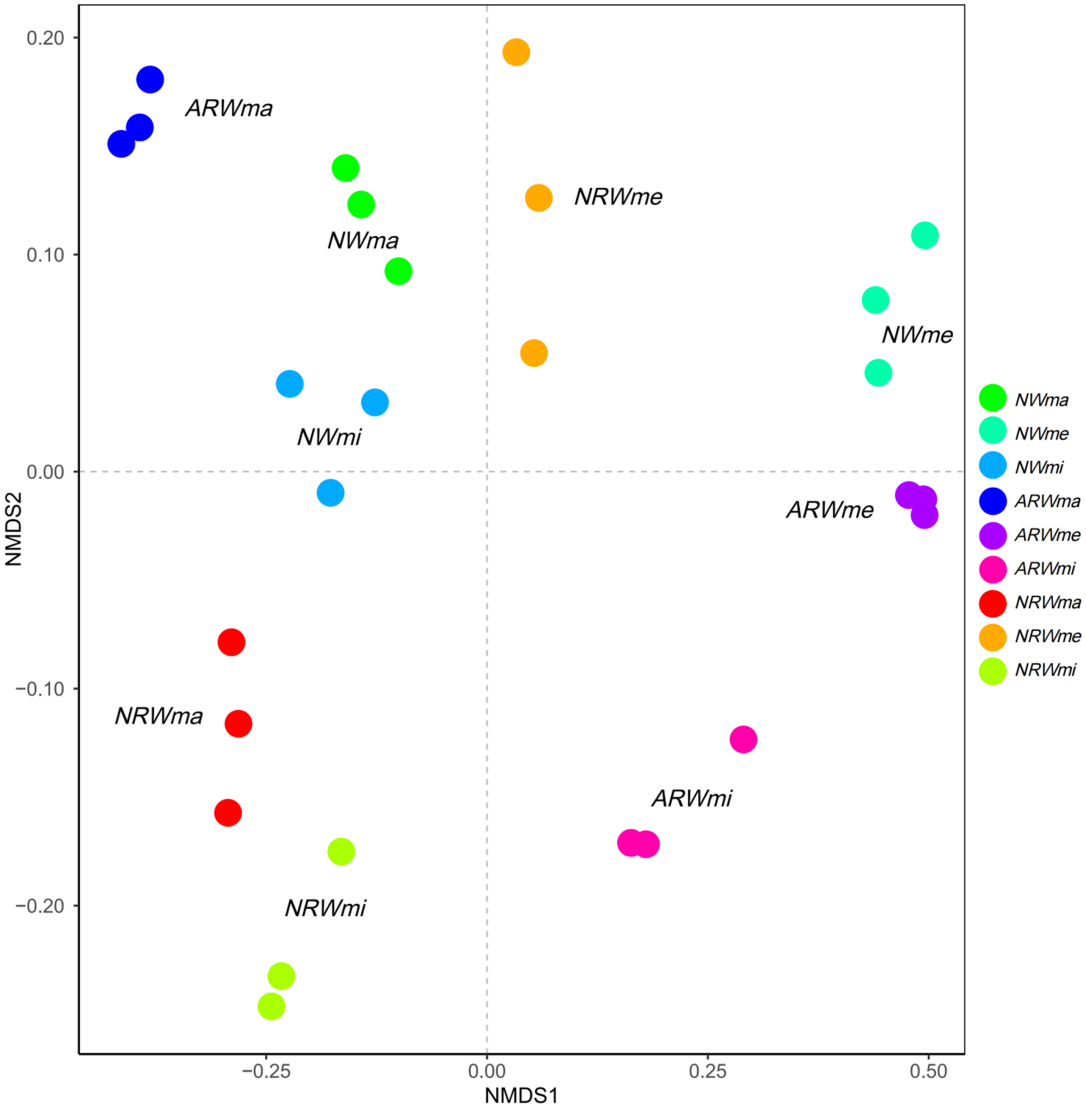
Nonmetric multidimensional scaling analysis based on Bray–Curtis distance at the operational taxonomic unit level (analysis of similarities test, r = 0.9961, *p* < 0.01). *NW*, natural wetland; *ARW*, artificially restored wetland; *NRW*, naturally restored wetland; *ma*, maximum plant Shannon index quadrat; *me*, median plant Shannon index quadrat; *mi*, minimum plant Shannon index quadrat.

As shown in Figure 1, the soil bacterial communities of the different treatments were obviously distinct (ANOSIM test, *r* = 0.9961, *p* < 0.01) at the OTU level, indicating that the bacterial community structure was significantly associated with plant diversity. The three quadrats of *ARW* were more decentralized, i.e., the bacterial community structure of *ARW* was more strongly associated with plant diversity. In addition, the *ARWme* bacterial community was more similar to the *ARWmi* bacterial community than the *ARWma* bacterial community. In both *NW* and *NRW*, the *ma* bacterial community was closer to the *mi* bacterial community than the *me* bacterial community. Additionally, compared to the *NRWme* bacterial community, the *ARWme* bacterial community was more similar to the *NWme* bacterial community.

### Soil bacterial community composition

**Figure 2:**
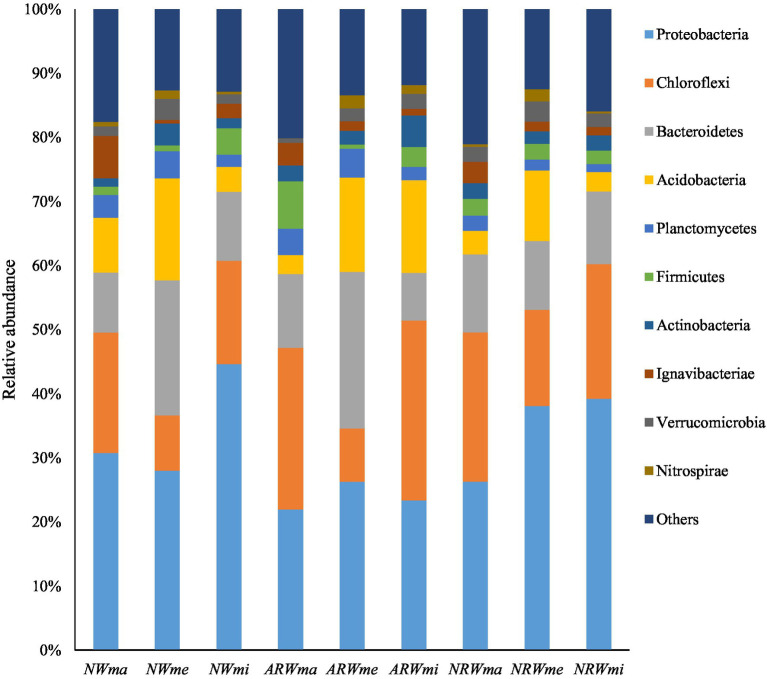
Top 10 phyla of the soil bacterial community. *NW*, natural wetland; *ARW*, artificially restored wetland; *NRW*, naturally restored wetland; *ma*, maximum plant Shannon index quadrat; *me*, median plant Shannon index quadrat; *mi*, minimum plant Shannon index quadrat.

At the 97% sequence similarity level, the entire soil microbial community (including bacteria and archaea) was identified to contain 47 phyla, 79 classes, 101 orders, 233 families, 645 genera, and 1,087 species in total. Excluding unassigned taxa, 35 bacterial phyla were obtained. The sum of relative abundances of the top four phyla was close to 70% of the total (Figure 2; Supplementary Table S1). The relative abundance of Proteobacteria was the highest, at above 20% in each sample, and it reached 44.65 and 21.95% in *NWmi* and *ARWma*, respectively. The relative abundance of Chloroflexi was significantly (*p* < 0.05) higher in *NWma* than that in *NWmi*, and the differences between *ARWma* and *ARWmi*, between *NRWma* and *NRWmi* were not significant (*p* > 0.05). The relative abundance of Bacteroidetes was significantly higher in *NWme* and *ARWme*, but did not significantly differ among *NRWma*, *NRWme*, and *NRWmi*.

**Figure 3:**
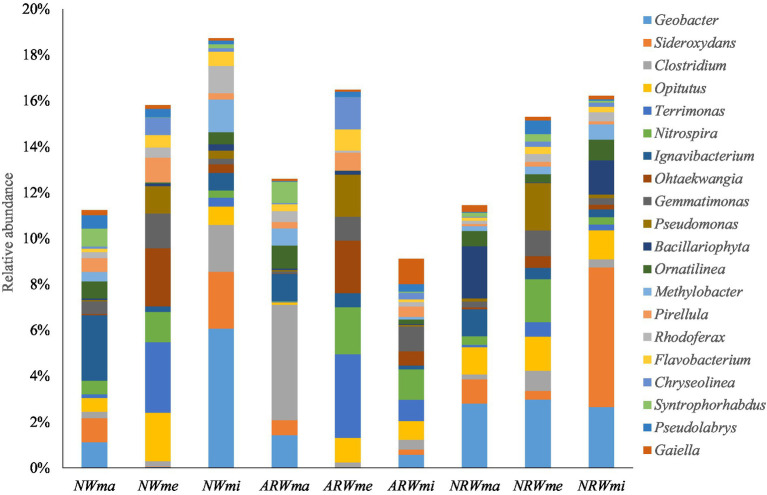
Top 20 genera of the soil bacterial community, excluding unassigned taxa. *NW*, natural wetland; *ARW*, artificially restored wetland; *NRW*, naturally restored wetland; *ma*, maximum plant Shannon index quadrat; *me*, median plant Shannon index quadrat; *mi*, minimum plant Shannon index quadrat.

At the genus level, excluding unassigned taxa, 626 bacterial genera were obtained, and the relative abundances of the top 20 genera are shown in Figure 3. The relative abundance of *Geobacter* (belonging to Proteobacteria) reached 6.07% in *NWmi* and close to 3% in *NRW* overall, but it was below 0.05% in both *NWme* and *ARWme*. The relative abundance of *Sideroxydans* (belonging to Proteobacteria) reached 6.09% in *NRWmi*, but was below 0.02% in both *NWme* and *ARWme*. The relative abundance of *Clostridium* (belonging to Firmicutes) reached 5.03 and 2.04% in *ARWma* and *NWmi*, respectively, but it was below 1% in the other samples. The relative abundance of *Opitutus* (belonging to Verrucomicrobia) reached 2.11% in *NWme*, but was only 0.1% in *ARWma*. The relative abundance of *Terrimonas* (belonging to Bacteroidetes) was above 3% in *NWme* and *ARWme*, but was below 1% in the other samples.

**Figure 4:**
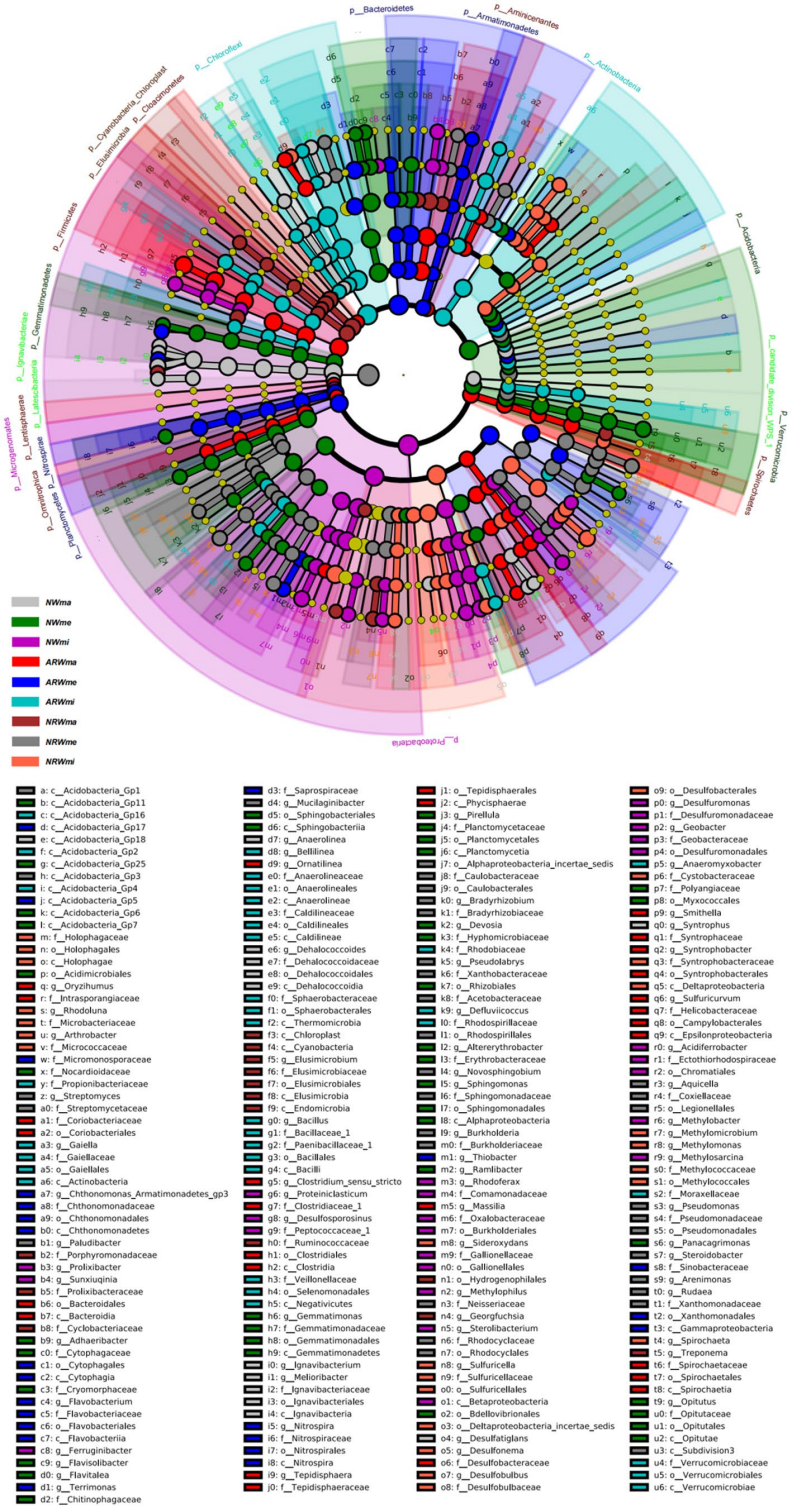
Linear discriminant analysis of effect size (LEfSe) analysis (LDA > 3, *p* < 0.05) of the soil bacterial community. The levels of phylum, class, order, family and genus are arranged from the outside to the inside. The yellow circle represents the taxa that did not significantly differ among the treatments. *NW*, natural wetland; *ARW*, artificially restored wetland; *NRW*, naturally restored wetland; *ma*, maximum plant Shannon index quadrat; *me*, median plant Shannon index quadrat; *mi*, minimum plant Shannon index quadrat.

Therefore, the soil bacterial community composition at the phylum and genus levels was significantly associated with plant diversity. Additionally, according to the LEfSe analysis (*p* < 0.05), when LDA > 2, the bacterial community biomarkers, including 32 phyla, 70 classes, 81 orders, 156 families, and 308 genera, significantly did differ among the different samples. When LDA > 3 (Figure 4), the bacterial community biomarkers included 22 phyla, 44 classes, 47 orders, 70 families, and 72 genera. There were 6 genera in *NWma*, 11 genera in *NWme*, 13 genera in *NWmi*, 8 genera in *ARWma*, 5 genera in *ARWme*, 5 genera in *ARWmi*, 3 genera in *NRWma*, 12 genera in *NRWme*, and 9 genera in *NRWmi*. For LDA > 4, the bacterial community biomarkers included 10 phyla, 18 classes, 18 orders, 11 families, and 5 genera, namely, *Ignavibacterium* (LDA = 4.106 in *NWma*), *Geobacter* (LDA = 4.398 in *NWmi*), *Clostridium* (LDA = 4.319 in *ARWma*), *Terrimonas* (LDA = 4.232 in *ARWme*), and *Sideroxydans* (LDA = 4.399 in *NRWmi*).

### Soil bacterial community diversity

The total number of OTUs obtained by clustering was 16, 894, and the number of shared OTUs was only 618. In each sample plot, the number of observed OTUs of *ma* was significantly (*p* < 0.05) higher than those of *me* and *mi* (Table 3), namely, soil bacterial species were more abundant under higher plant diversity. Overall, the bacterial Chao1 index and Shannon index of *ma* were significantly higher than those of *me* and *mi*, i.e., the higher the plant Shannon index, the higher the bacterial Shannon index. However, the difference in bacterial Shannon index between *me* and *mi* was not significant (*p* > 0.05). The difference in bacterial Simpson’s index values between *ma* and *me* was not significant, and in *NRW*, the *mi* was not significantly different.

**Table 3:** Diversity indexes of the soil bacterial community.

Index	Quadrat	Sample plot								
		*NW*			*ARW*			*NRW*		
Observed	*ma*	b	4,604 ± 67	a	c	4,053 ± 82	a	a	5,083 ± 120	a
	*me*	a	3,353 ± 56	c	a	3,213 ± 121	b	a	3,470 ± 171	b
	*mi*	a	3,738 ± 218	b	a	3,428 ± 277	b	a	3,788 ± 439	b
Chao1	*ma*	a	6638.144 ± 166.951	a	b	5229.186 ± 226.644	a	a	6787.141 ± 143.728	a
	*me*	ab	4560.236 ± 211.105	c	b	4329.303 ± 117.189	b	a	4863.193 ± 197.837	b
	*mi*	a	5335.269 ± 297.842	b	a	4658.387 ± 286.579	b	a	5211.138 ± 623.290	b
Shannon	*ma*	a	6.946 ± 0.032	a	b	6.812 ± 0.086	a	ab	6.907 ± 0.033	a
	*me*	a	6.559 ± 0.056	b	b	6.400 ± 0.043	b	a	6.547 ± 0.081	ab
	*mi*	a	6.431 ± 0.223	b	a	6.175 ± 0.249	b	a	6.426 ± 0.366	b
Simpson	*ma*	a	0.997 ± 0.000	a	b	0.995 ± 0.000	a	b	0.994 ± 0.001	a
	*me*	a	0.995 ± 0.001	ab	a	0.994 ± 0.000	a	a	0.995 ± 0.001	a
	*mi*	a	0.993 ± 0.002	b	b	0.977 ± 0.009	b	a	0.990 ± 0.004	a

Data represent the means ± standard deviations. Analysis of variance (with Duncan’s multiple comparison test) was used to test the significance of differences. Values labeled with different lowercase letters were significant different (*p* < 0.05). The left letters correspond to comparisons of the differences among the three sample plots, and the right letters correspond to comparisons of the differences among the three quadrats within each sample plot. NW, natural wetland; ARW, artificially restored wetland; NRW, naturally restored wetland; ma, maximum plant Shannon index quadrat; me, median plant Shannon index quadrat; mi, minimum plant Shannon index quadrat.

Among the three sample plots, *NRWma* OTUs significantly outnumbered *NWma* and *ARWma* OTUs, and the differences in OTU number between *me* and *mi* were not significant. The *ARWma* bacterial Chao1 index value was significantly lower than the *NWma* and *NRWma* bacterial Chao1 index values, and that of *ARWme* was significantly lower than that of *NRWme*, however, the difference was not significant among *NWmi*, *ARWmi*, and *NRWmi*. The bacterial Shannon index was lower in *ARW*, and the difference was not significant among *NWmi*, *ARWmi*, and *NRWmi*. The bacterial Simpson’s index value among *NWme*, *ARWme* and *NRWme* showed no significant difference, but it was significantly higher and lower in *NWma* and *ARWmi*, respectively.

**Figure 5:**
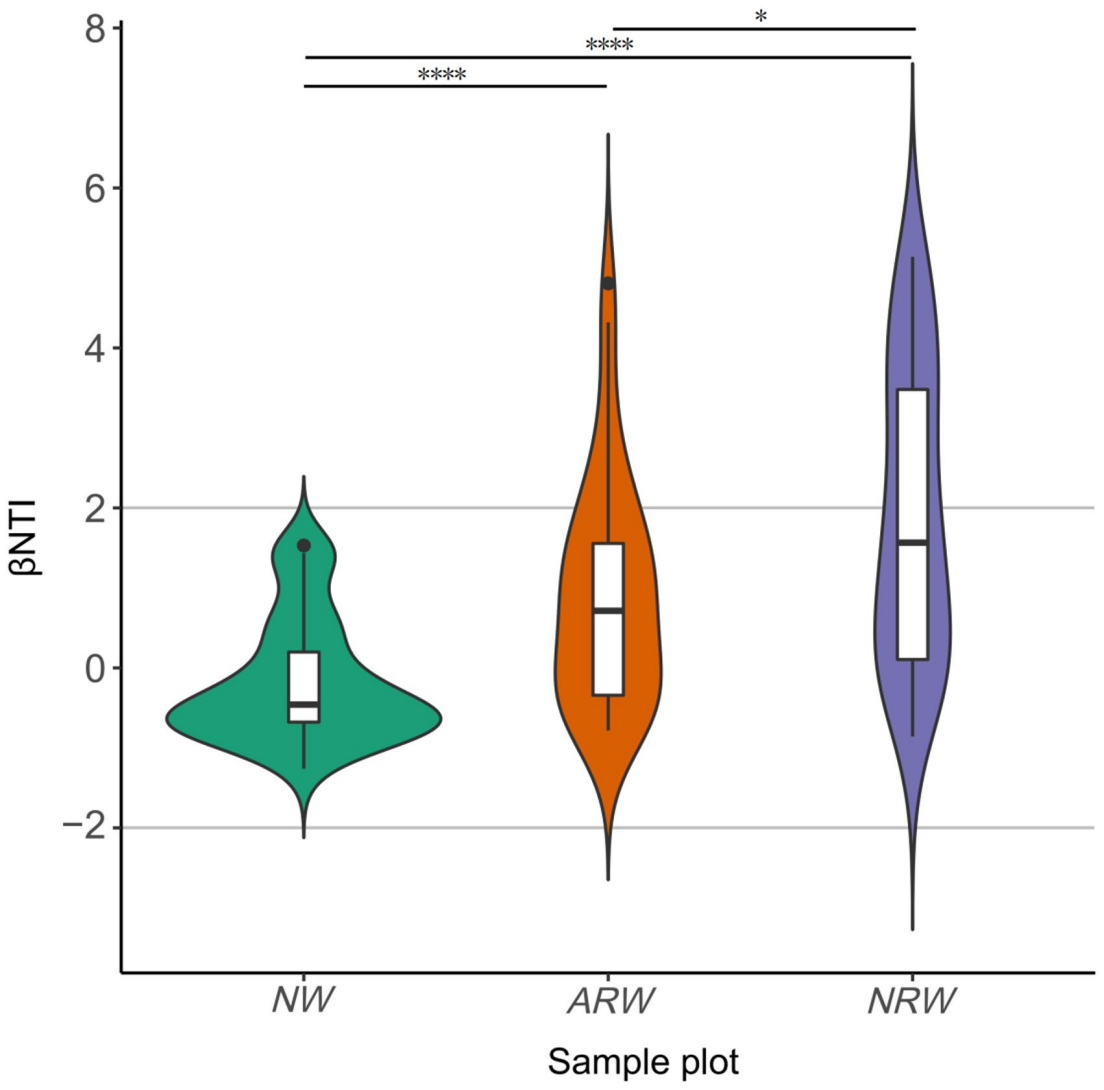
β–nearest taxon index (βNTI) score distribution of soil bacterial communities of *NW*, *ARW*, and *NRW*. βNTI, β–nearest taxon index; *NW*, natural wetland; *ARW*, artificially restored wetland; *NRW*, naturally restored wetland. The significance measured using Bray–Curtis dissimilarity, ^*^p < 0.05, ^****^p < 0.0001.

The difference in the phylogenetic diversity of the soil bacterial community between *ARW* and *NRW* was significant (*p* < 0.05), and both of them exhibited significant (*p* < 0.0001) differences from that of *NW* (Figure 5). All the |βNTI| scores of *NW* were below two (Supplementary Table S2), indicating stochastic processes largely determined the phylogenetic diversity of the bacterial community. Among *ARW* |βNTI| scores, 80.56% were below two, which indicated the dominant role of stochastic processes. However, the |βNTI| scores of *NRW* below and above two accounted for 52.78 and 47.22% of scores, respectively, indicating a balance between deterministic and stochastic processes. Thus, the stochastic processes underlying the formation of the phylogenetic diversity of bacterial communities were less important in *NRW*.

### Correlations among soil physicochemical, plant diversity, and soil bacterial diversity indexes

The Pearson correlation analysis results are shown in Table 4. The bacterial Shannon index was significantly (*p* < 0.01) positively correlated with the plant Shannon index and the Pielou index and significantly (*p* < 0.01) negatively correlated with the plant Simpson’s index. Both the positive correlation between the Chao1 index and the Pielou index and the positive correlation between the bacterial Simpson’s index and the plant Shannon index were significant (*p* < 0.05).

**Table 4:** Correlations among bacterial diversity indexes, plant diversity indexes, and soil physicochemical indexes.

		Bacterial diversity index			Plant diversity index		
		Chao1	Shannon	Simpson	Shannon	Pielou	Simpson
Plant diversity index	Shannon	0.565	0.917^**^	0.701^*^			
	Pielou	0.695^*^	0.923^**^	0.593	0.930^**^		
	Simpson	−0.617	−0.857^**^	−0.578	−0.938^**^	−0.931^**^	
Soil physico-chemical index	MC	−0.255	−0.717^*^	−0.713^*^	−0.895^**^	−0.746^*^	0.857^**^
	BD	0.147	0.644	0.726^*^	0.865^**^	0.685^*^	−0.745^*^
	pH	−0.053	−0.499	−0.511	−0.734^*^	−0.652	0.797^*^
	TOC	0.347	0.752^*^	0.654	0.903^**^	0.728^*^	−0.793^*^
	TN	0.364	0.779^*^	0.585	0.942^**^	0.847^**^	−0.849^**^
	AN	0.728^*^	0.874^**^	0.558	0.864^**^	0.959^**^	−0.890^**^
	TP	0.339	0.771^*^	0.643	0.890^**^	0.810^**^	−0.893^**^
	AP	0.295	0.683^*^	0.718^*^	0.793^*^	0.549	−0.642
	TK	−0.006	0.379	0.293	0.491	0.397	−0.545
	AK	−0.015	0.382	0.502	0.644	0.504	−0.701^*^

MC, moisture content; BD, bulk density; TOC, total organic C; TN, total N; AN, alkali–hydrolyzed N; TP, total P; AP, available P; TK, total K; AK, available K. ^*^p < 0.05, ^**^p < 0.01.

The bacterial Shannon index was significantly (*p* < 0.05) correlated with soil MC, TOC, TN, and P, and the correlation with AN was significantly (*p* < 0.01) positive. The Chao1 index was significantly (*p* < 0.05) positively correlated with AN. The bacterial Simpson’s index was significantly (*p* < 0.05) negatively correlated with MC, and it was significantly (*p* < 0.05) positively correlated with BD and AP. The bacterial diversity indexes were not significantly (*p* > 0.05) correlated with pH, TK, and AK.

The plant Shannon index was significantly (*p* < 0.05 or *p* < 0.01) correlated with the soil physicochemical indexes except the soil K content. The Pielou index was not significantly correlated with pH, AP, and K. Additionally, the plant Simpson’s index was not significantly correlated with AP and TK. MC and pH were positively correlated with the plant Shannon index and the Pielou index, but they were negatively correlated with the plant Simpson’s index.

### Soil bacterial community function prediction

**Figure 6:**
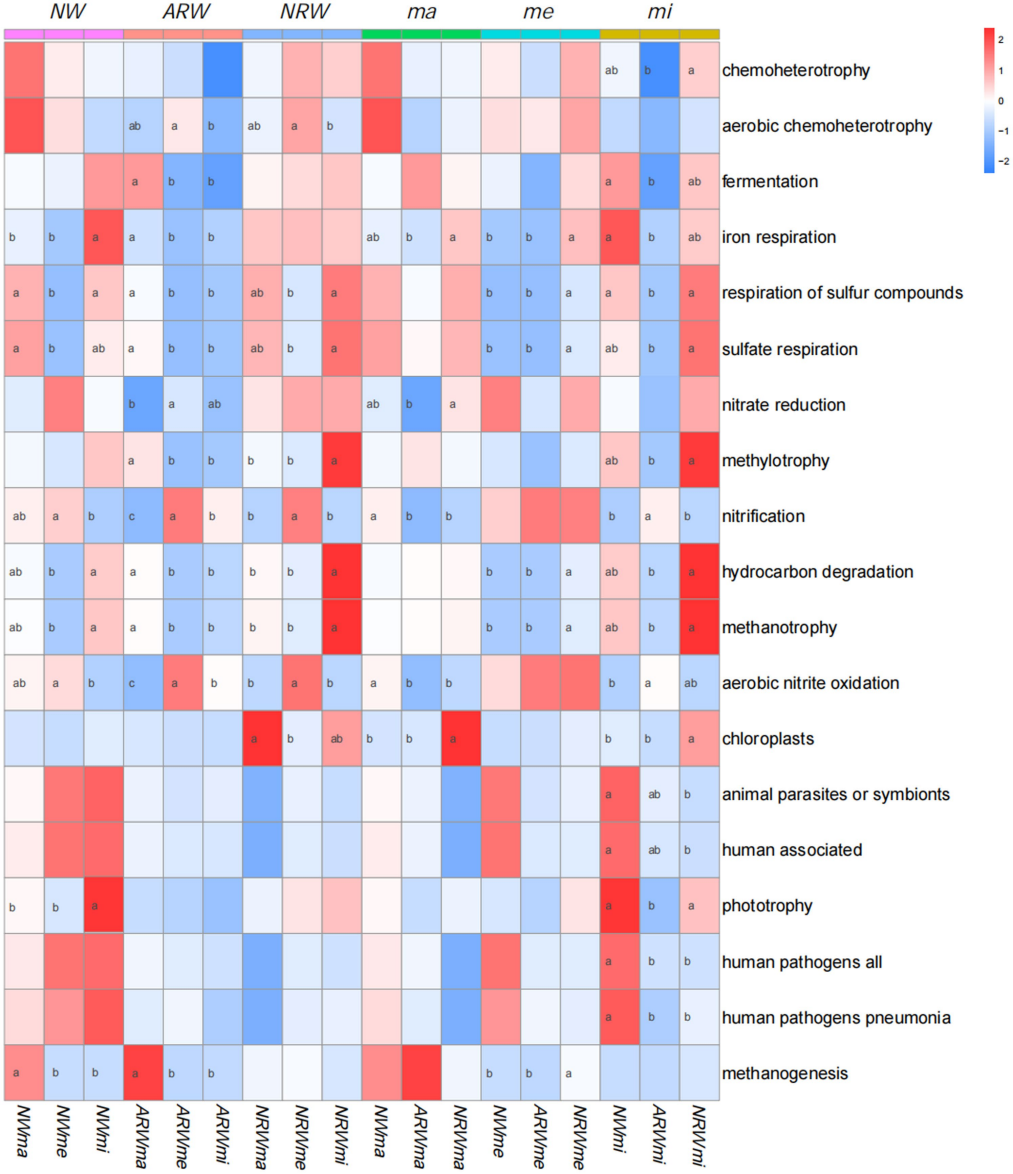
Functional prediction using FAPROTAX. The darker the red color, the higher the relative proportion is, and the darker the blue color, the lower the relative proportion is. Analysis of variance (with Duncan’s multiple comparison test) was used to test the significance of differences. Values labeled with different lowercase letters were significant different (*p* < 0.05). *NW*, natural wetland; *ARW*, artificially restored wetland; *NRW*, naturally restored wetland; *ma*, maximum plant Shannon index quadrat; *me*, median plant Shannon index quadrat; *mi*, minimum plant Shannon index quadrat.

FAPROTAX prediction identified 71 functional groups, and the relative proportions of the 19 functional groups were all above 1% (Figure 6). Chemoheterotrophy, aerobic chemoheterotrophy, fermentation, and iron respiration groups contained more OTUs, and the other functional groups were related to soil S, N, and C cycles. The relative proportion of chemoheterotrophic bacteria among the three sampled quadrats in each sample plot was not significantly (*p* > 0.05) different, and only that of *NRWmi* was significantly (*p* < 0.05) higher than that of *ARWmi*, but neither of them was significantly different from that of *NWmi*. Additionally, plant diversity was not significantly associated with chemoheterotrophy. The relative proportions of aerobic chemoheterotrophic bacteria in *ARWme* and *NRWme* were significantly higher than those in *ARWmi* and *NRWmi*, but there were non-significant differences compared to those in *ARWma* and *NRWma*, respectively.

The relative proportion of S cycle-related functional groups, such as respiration of S compounds and sulfate respiration, was lower in *NWme*, *ARWme*, and *NRWme*, and there was no significant difference in this relative proportion between *ma* and *mi* of each sample plot. The difference in this relative proportion among *NWma*, *ARWma*, and *NRWma* was not significant. That of *NRWme* was significantly higher than those of *NWme* and *ARWme*, and that of *ARWmi* was lower than those of *NWmi* and *NRWmi*.

The relative proportion of N cycle–related functional groups, such as nitrate reduction, nitrification, and aerobic nitrite oxidation, was higher in *NWme*, *ARWme*, and *NRWme* than in the other samples, and that in *ARWme* was significantly higher than that in *ARWma*. The difference in this relative proportion among *NWme*, *ARWme*, and *NRWme* was not significant. The relative proportion of nitrate-reducing bacteria in *NRWma* was significantly higher than that in *ARWma*. However, the relative proportions of nitrification and aerobic nitrite oxidation in *NRWma* and *ARWma* were significantly lower than that in *NWma*, and that in *ARWmi* was significantly higher than that in *NWmi*.

The relative proportion of C cycle-related functional groups, such as methylotrophy, hydrocarbon degradation, methanotrophy, and methanogenesis, was significantly higher in *ARWma* than those in *ARWme* and *ARWmi*. The relative proportions of hydrocarbon degradation and methanotrophy were higher in *NWmi* than those in *NWme*, and the *NWma* had a significantly higher relative proportion of methanogenesis functional groups. Except for the methanogenesis functional group, *NRWmi* had a significantly higher relative proportion of C cycle–related functional groups than *NRWma* and *NRWme*. Among the three sample plots, the difference in the relative proportion was not significant among *NWma*, *ARWma*, and *NRWma*, and it was significantly higher in *NRWme* and *NRWmi* than in the other samples, respectively.

## Discussion

### Association of soil bacterial community diversity with plant diversity

The Shannon index is one of the most frequently used biodiversity indexes (Palaghianu, 2016). It was used to assess plant diversity in this study, and the maximum, median, and minimum Shannon index values were used to describe the plant diversity characteristics of the three sample plots, respectively. Based on the description and comparison of diversity indexes (Das et al., 2006; Palaghianu, 2016), the Pielou index and the plant Simpson’s index were significantly (*p* < 0.01) positively and negatively correlated with the plant Shannon index, respectively in this study. Thus, when the dominance of *C. appendiculata* increased in *Carex* tussock wetland, the plant Simpson’s index increased accordingly, but the Pielou index and the plant Shannon index instead decreased.

Kim et al. (2022) found that structurally and functionally distinct microbial communities develop under different plant species in wetlands, suggesting that it is important to consider the diversity of plant species, along with abiotic factors, when investigating the abundance and coexistence of different microbial species. Calheiros et al. (2009) demonstrated that high plant richness could increase bacterial abundance and community structure profiles in wetlands. Li et al. (2021) reported that wetland plants could enhance the soil microbial diversity. In *Carex* tussock wetland, the soil bacterial Shannon index was also significantly positively correlated with the plant Shannon index in this study, that is, a plant diversity increase could be associated with an increase in the diversity of soil bacteria.

The higher soil MC and the lower BD were consistent with surface ponding in the quadrats of *Carex* tussock wetland in this study. In these quadrats, the plant species and quantity of companion species decreased. Water is a key driver of plant diversity and community structure in wetland ecosystems, and it can change the vegetation and biomass (Li et al., 2015; Feng et al., 2020; Shan et al., 2020). In particular, in *Carex* tussock wetland, the growth and physiology of plants and species diversity respond to water level fluctuations (Wang et al., 2016; Zhang et al., 2020). Meanwhile, soil bacterial community structure is also strongly shaped by water content (Sui et al., 2021). Both the plant and bacterial Shannon indexes decreased in the *mi* with surface ponding in this study. Soil pH is a major factor driving variation in bacterial diversity and community structure (Kang et al., 2021; Mod et al., 2021). In this study, the soil pH was negatively correlated with the bacterial diversity indexes, but the correlation was not significant; this may be owing to the small range of pH values observed.

Wang et al. (2019) and Wang M. et al. (2021) inferred that SOC, TN, and TP contents in *Carex* tussock wetlands were the main environmental factors affecting the plant community structure, abundance, and diversity. Soil nutrient content was positively associated with plant diversity. The present study also found that the soil nutrient (except for K) content was significantly (*p* < 0.05 or *p* < 0.01) positively correlated with the plant Shannon index. Chen et al. (2021) reported that when plant diversity is low, litter is reduced, and the nutrients available for soil microorganisms are reduced accordingly. This leads to the reduction of the microbial population and community diversity. The results of the present study also support this conclusion.

Soil microbial communities are closely related to plant diversity and composition, and changes in plant diversity and composition can affect bacterial community composition (Zak et al., 2003; Shi et al., 2021). In the present study, the soil bacterial communities under the different levels of plant diversity were obviously separated at the OTU level, indicating that the composition of the soil bacterial community was associated with plant diversity. Proteobacteria, Chloroflexi, and Bacteroidetes were shown to be the dominant phyla in the soil of plateau wetland and coastal wetland in China (An et al., 2019). These phyla also had higher relative abundance in the soil bacterial community in the present study, and under different plant diversity and composition conditions, the relative abundance significantly differed. Meanwhile, *Ignavibacterium*, *Geobacter*, *Clostridium*, and other genera, also significantly differed in relative abundance. In *Carex* tussock wetland, soil microbial community heterogeneity is promoted by variation among micro–habitats (Xu et al., 2004). Therefore, under the different levels of plant diversity and composition, soil bacterial community structure and species composition differed.

### Association of the soil bacterial community function with plant diversity

FAPROTAX focuses on marine and lacustrine biogeochemistry, especially the S, N, and C cycles (Louca et al., 2016). In this study, chemoheterotrophy, aerobic chemoheterotrophy, and fermentation groups were represented at higher relative proportions, but were not significantly associated with plant diversity in *NW*; meanwhile, the relative proportions of chemoheterotrophic bacteria were not significantly different between *ARW* and *NRW*. Wetland ecosystems often experience year-round or seasonal flooding, resulting in poor soil aeration (Hu et al., 2021). The relative proportions of aerobic chemoheterotrophic bacteria in *ARWmi* and *NRWmi* were lower than those in *ARWme* and *NRWme*, although they were both non–significantly different from *ARWma* and *NRWma*, respectively, this might be owing to the water level control and higher MC in *mi*.

Soil S, N, and C are known to have important interactions in wetland ecosystems (Liu et al., 2021; Wang Q. et al., 2021). The S cycle in a constructed wetland microcosm was found to have electron mediating ability between C and N cycles (Guo et al., 2020). This study also showed that the S, N, and C cycles are interrelated, and the relative proportions of bacteria associated with S and C cycles were lower, though that of N cycle bacteria was higher. Bacteria (Proteobacteria, Chloroflexi, Nitrospirae, etc.) and archaea participate in the chemical cycle of wetland soil (Mellado and Vera, 2021). Liu et al. (2022) reported that the S and N cycles were significantly affected by wetland plant composition and coverage with the participation of bacteria. The results of the present study also indicated that the soil S, N, and C cycles were associated with plant diversity, likely through altering the microbial community.

Wetlands are an important source of methane emissions (Peng et al., 2022). Wetland plants can influence methane emissions by altering methane production, consumption and transport in the wetland soil (Koelbener et al., 2010), and the interaction between wetland plants and soil microbes affects the soil C cycle (Schmid et al., 2021). In the present study, as shown in Figure 6, higher wetland plant diversity appears to increase methane emissions. In the *me* and *mi* samples, the relative proportion of C cycle-related functional groups in *NRW* was significantly higher than that in *ARW*. Therefore, the *NRW* appeared to have enhanced soil C cycles.

### *Carex* tussock wetland restoration suggestion

Yu et al. (2010) reported that tussocks facilitate the establishment of plant species inside them and increase both diversity and reproduction. Thus, tussocks are of great importance to the stability of wetland ecosystems. Wang et al. (2018) noted that tussock–forming *Carex* could be the preferred species for vegetation restoration of degraded peat bogs in view of their strong promotion of species diversity. Therefore, the restoration of *Carex* tussock wetland is very important for the protection of entire wetland ecosystems.

The genus *Carex* is comprised of more than 2,000 species (Bernard, 1990), however, only a few species can form tussocks, and *Carex* tussock formation requires a long time (Wang et al., 2018; Zhang et al., 2020; Qi et al., 2021). Qi et al. (2021) previously reported that root cloning and transplanting along with hydrological regulation can be used to accelerate plant diversity restoration in *Carex* tussock wetland. In the present study, on the whole, there was no significant difference in plant diversity among *ARW*, *NRW* and *NW* after 10 years of restoration, indicating that both types of restoration methods can achieve the desired results. However, in *NRW*, there were also mesophytes, while the species of companion plants in *ARW* were closer to those in *NW*, both of which were dominated by hygrophytes.

Soil microbial communities change along with the process of vegetation restoration in wetlands (Bonetti et al., 2021; Jeong and Kim, 2021). The present study indicated that there were significant differences in the phylogenetic diversity of soil bacterial communities among *NW*, *ARW*, and *NRW*. Stochastic processes played a significantly greater role in shaping the phylogenetic diversity of *ARW* relative to *NRW*. Therefore, *ARW* were closer to *NW* in terms of restoration indicators considering vegetation composition and the microbial community, such that the techniques used in *ARW* could be recommended for the restoration of *Carex* tussock wetlands.

## Conclusion

In *Carex* tussock wetlands, different vegetation restoration methods did not differ in their effect on plant diversity, but did appear to change the plant species composition. The soil bacterial community compositions were also significantly different under different plant diversity conditions, and the relative abundance of phyla and genera was significantly associated with plant diversity. Surface ponding appeared to lead to an increase in soil MC, a decrease in BD and nutrient content, and decreases in both plant diversity and bacterial diversity. The phylogenetic diversity of bacterial communities in restored wetlands was significantly (*p* < 0.0001) different from that of *NW*, and compared to *NRW*, stochastic processes had a more similar role in shaping the phylogenetic diversity of *ARW* relative to *NW*. Different levels of plant diversity exhibited different relative proportions of functional groups comprising the bacterial community, especially affecting the relative proportions of bacteria affecting the wetland soil S, N, and C cycles. Higher plant diversity was likely associated with increased *Carex* tussock wetland soil methane emissions. According to both the plant and soil bacterial communities rehabilitated, the method of root cloning and transplantation coupled with hydrological regulation is recommended for *Carex* tussock wetland restoration.

## Data availability statement

The datasets presented in this study can be found in online repositories. The names of the repository/repositories and accession number(s) can be found in the article/Supplementary material.

## Author contributions

YL and CS designed and performed the experiment and prepared this manuscript. DW and LW designed this experiment. JD, NX, and LJ helped to perform the experiment and process the data. All coauthors contributed to manuscript editing. All authors have read and agreed to the published version of the manuscript.

## Funding

This work was supported by National Key Research and Development Plan of China (2022YFD1601102), Strategic Priority Research Program of the Chinese Academy of Sciences (XDA28130200), Beijing Academy of Agricultural and Forestry Sciences Youth Fund (QNJJ202214), Beijing Postdoctoral Fund (Artificial sponge soil construction and its water regulation mechanism), Postdoctoral Research Fund of Beijing Academy of Agricultural and Forestry Sciences (2020-ZZ-026), and Natural Science Foundation of Heilongjiang Province (LH2019D014 and LH2022C053).

## Conflict of interest

The authors declare that the research was conducted in the absence of any commercial or financial relationships that could be construed as a potential conflict of interest.

## Publisher’s note

All claims expressed in this article are solely those of the authors and do not necessarily represent those of their affiliated organizations, or those of the publisher, the editors and the reviewers. Any product that may be evaluated in this article, or claim that may be made by its manufacturer, is not guaranteed or endorsed by the publisher.

## Footnotes

^1^ https://huttenhower.sph.harvard.edu/galaxy/
