# Supplementary figures and images for: Associations of soil bacterial diversity and function with plant diversity in Carex tussock wetland
Source: Front Microbiol. 2023 Mar 1;14:1142052. doi: 10.3389/fmicb.2023.1142052 (PMC10115198; doi:10.3389/fmicb.2023.1142052)

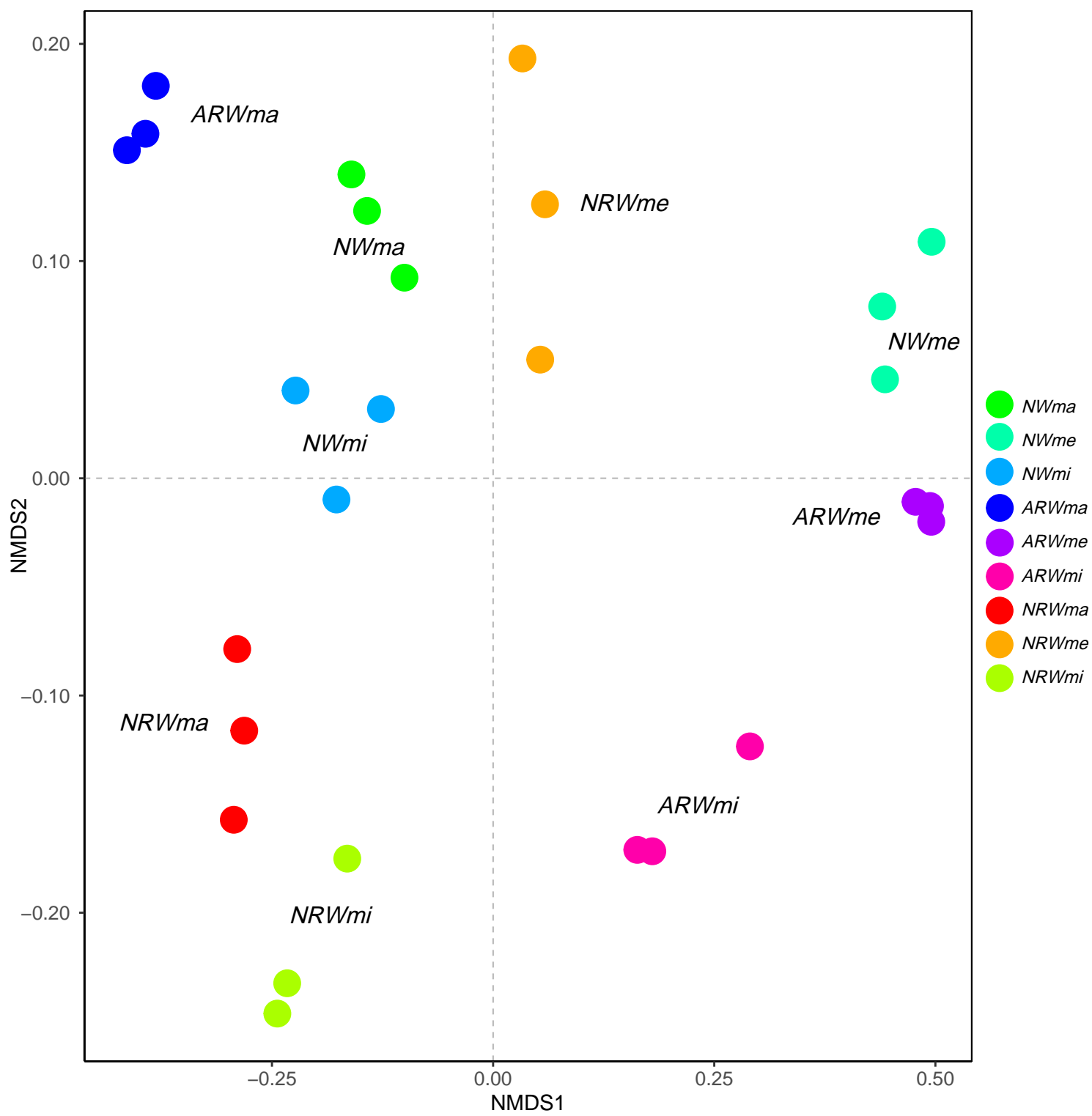

Supplement: Supplementary file 1 [file Data_Sheet_1.PDF]

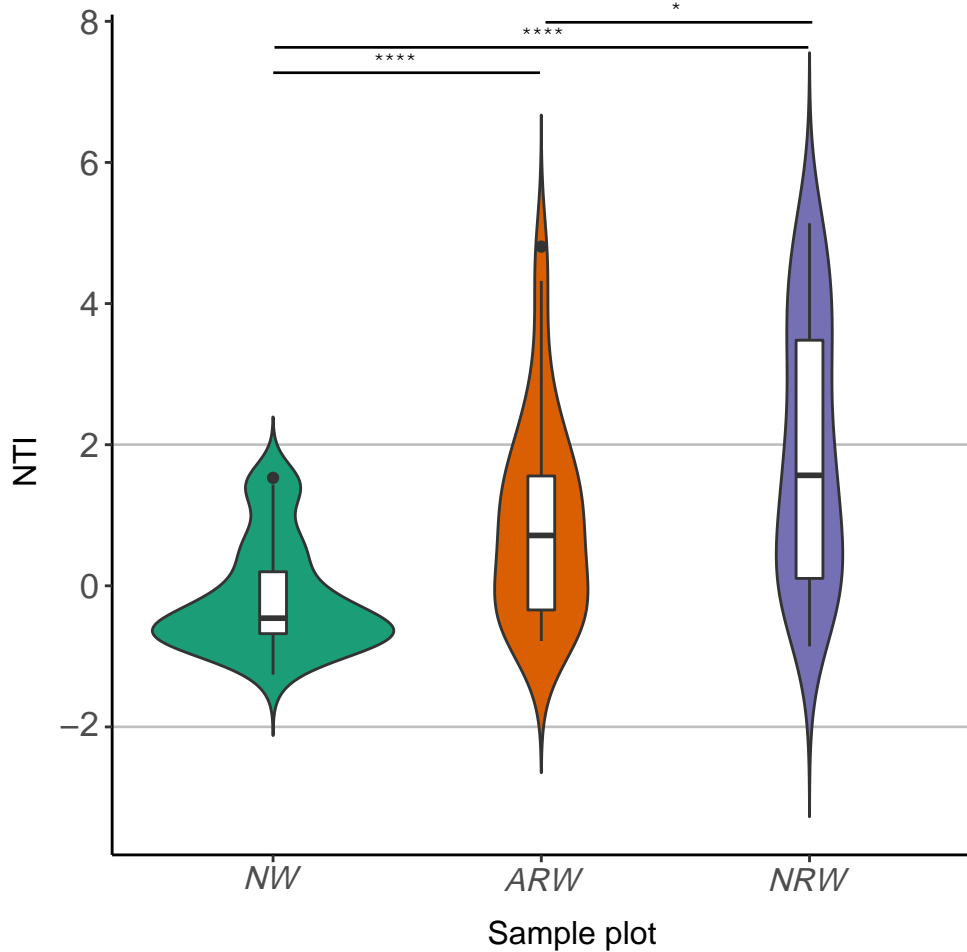

Supplement: Supplementary file 3 [file Data_Sheet_3.PDF]

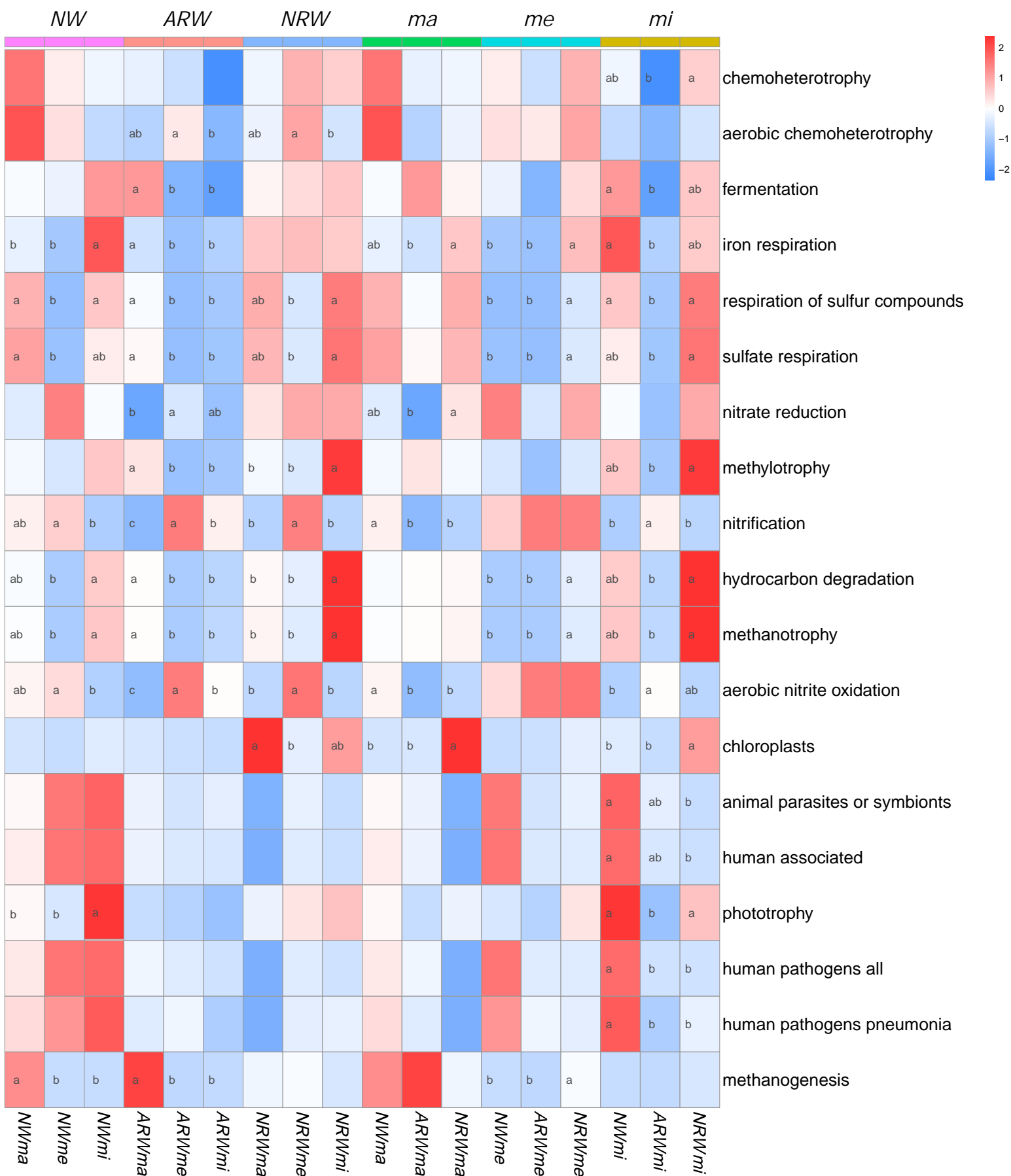

Supplement: Supplementary file 4 [file Data_Sheet_4.PDF]
