# Supplementary material for: Associations of soil bacterial diversity and function with plant diversity in Carex tussock wetland
Source: Front Microbiol. 2023 Mar 1;14:1142052. doi: 10.3389/fmicb.2023.1142052 (PMC10115198; doi:10.3389/fmicb.2023.1142052)
Supplement: Supplementary file 2 [file Data_Sheet_2.PDF]

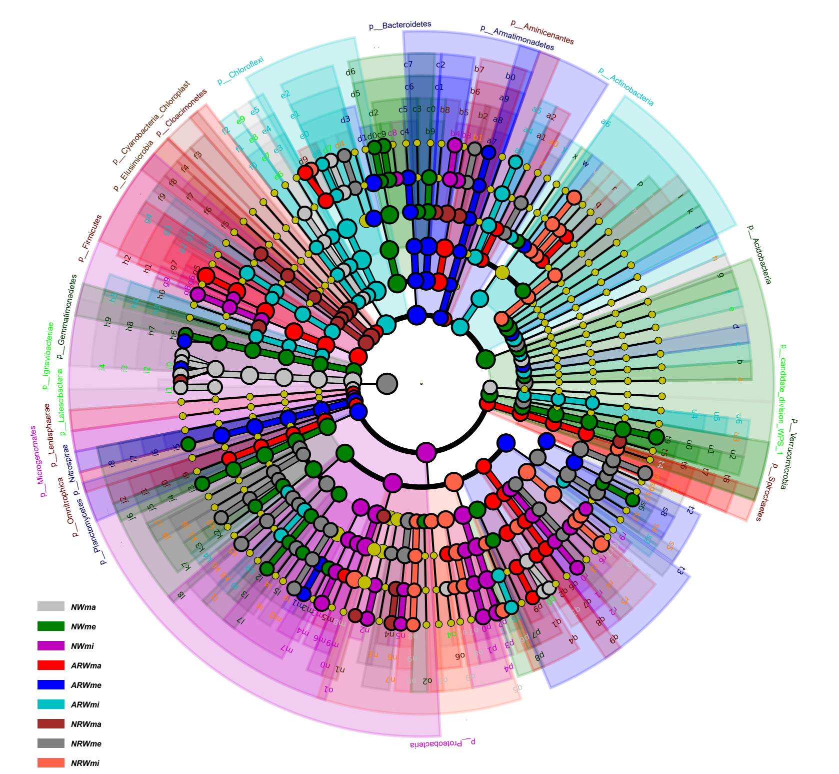

a: c__Acidobacteria_Gp1
b: c__Acidobacteria_Gp11
c: c__Acidobacteria_Gp16
d: c__Acidobacteria_Gp17
e: c__Acidobacteria_Gp18
f: c__Acidobacteria_Gp2
g: c__Acidobacteria_Gp25
h: c__Acidobacteria_Gp3
i: c__Acidobacteria_Gp4
j: c__Acidobacteria_Gp5
k: c__Acidobacteria_Gp6
l: c__Acidobacteria_Gp7
m: f__Holophagaceae
n: o__Holophagales
o: c__Holophagae
p: o__Acidimicrobiales
q: g__Oryzihumus
r: f__Intrasporangiaceae
s: g__Rhodoluna
t: f__Microbacteriaceae
u: g__Arthrobacter
v: f__Micrococcaceae
w: f__Micromonosporaceae
x: f__Nocardioidaceae
y: f__Propionibacteriaceae
z: g__Streptomyces
a0: f__Streptomycetaceae
a1: f__Coriobacteriaceae
a2: o__Coriobacteriales
a3: g__Gaiella
a4: f__Gaiellaceae
a5: o__Gaiellales
a6: c__Actinobacteria
a7: g__Chthonomonas_Armatimonadetes_gp3
a8: f__Chthonomonadaceae
a9: o__Chthonomonadales
b0: c__Chthonomonadetes
b1: g__Paludibacter
b2: f__Porphyromonadaceae
b3: g__Prolixibacter
b4: g__Sunxiuqinia
b5: f__Prolixibacteraceae
b6: o__Bacteroidales
b7: c__Bacteroidia
b8: f__Cyclobacteriaceae
b9: g__Adhaeribacter
c0: f__Cytophagaceae
c1: o__Cytophagales
c2: c__Cytophagia
c3: f__Cryomorphaceae
c4: g__Flavobacterium
c5: f__Flavobacteriaceae
c6: o__Flavobacteriales
c7: c__Flavobacteriia
c8: g__Ferruginibacter
c9: g__Flavisolibacter
d0: g__Flavitalea
d1: g__Terrimonas
d2: f__Chitinophagaceae
d3: f__Saprospiraceae
d4: g__Mucilaginibacter
d5: o__Sphingobacteriales
d6: c__Sphingobacteriia
d7: g__Anaerolinea
d8: g__Bellilinea
d9: g__Ornatilinea
e0: f__Anaerolineaceae
e1: o__Anaerolineales
e2: c__Anaerolineae
e3: f__Caldilineaceae
e4: o__Caldilineales
e5: c__Caldilineae
e6: g__Dehalococcoides
e7: f__Dehalococcoidaceae
e8: o__Dehalococcoidales
e9: c__Dehalococcoidia
f0: f__Sphaerobacteraceae
f1: o__Sphaerobacterales
f2: c__Thermomicrobia
f3: c__Chloroplast
f4: c__Cyanobacteria
f5: g__Elusimicrobium
f6: f__Elusimicrobiaceae
f7: o__Elusimicrobiales
f8: c__Elusimicrobia
f9: c__Endomicrobia
g0: g__Bacillus
g1: f__Bacillaceae_1
g2: f__Paenibacillaceae_1
g3: o__Bacillales
g4: c__Bacilli
g5: g__Clostridium_sensu_stricto
g6: g__Proteiniclasticum
g7: f__Clostridiaceae_1
g8: g__Desulfosporosinus
g9: f__Peptococcaceae_1
h0: f__Ruminococcaceae
h1: o__Clostridiales
h2: c__Clostridia
h3: f__Veillonellaceae
h4: o__Selenomonadales
h5: c__Negativicutes
h6: g__Gemmatimonas
h7: f__Gemmatimonadaceae
h8: o__Gemmatimonadales
h9: c__Gemmatimonadetes
i0: g__Ignavibacterium
i1: g__Melioribacter
i2: f__Ignavibacteriaceae
i3: o__Ignavibacteriales
i4: c__Ignavibacteria
i5: g__Nitrospira
i6: f__Nitrospiraceae
i7: o__Nitrospirales
i8: c__Nitrospira
i9: g__Tepidisphaera
j0: f__Tepidisphaeraceae
j1: o__Tepidisphaerales
j2: c__Phycisphaerae
j3: g__Pirellula
j4: f__Planctomycetaceae
j5: o__Planctomycetales
j6: c__Planctomycetia
j7: o__Alphaproteobacteria_incertae_sedis
j8: f__Caulobacteraceae
j9: o__Caulobacterales
k0: g__Bradyrhizobium
k1: f__Bradyrhizobiaceae
k2: g__Devosia
k3: f__Hyphomicrobiaceae
k4: f__Rhodobiaceae
k5: g__Pseudolabrys
k6: f__Xanthobacteraceae
k7: o__Rhizobiales
k8: f__Acetobacteraceae
k9: g__Defluviicoccus
l0: f__Rhodospirillaceae
l1: o__Rhodospirillales
l2: g__Altererythrobacter
l3: f__Erythrobacteraceae
l4: g__Novosphingobium
l5: g__Sphingomonas
l6: f__Sphingomonadaceae
l7: o__Sphingomonadales
l8: c__Alphaproteobacteria
l9: g__Burkholderia
m0: f__Burkholderiaceae
m1: g__Thiobacter
m2: g__Ramlibacter
m3: g__Rhodoferax
m4: f__Comamonadaceae
m5: g__Massilia
m6: f__Oxalobacteraceae
m7: o__Burkholderiales
m8: g__Sideroxydans
m9: f__Gallionellaceae
n0: o__Gallionellales
n1: o__Hydrogenophilales
n2: g__Methylophilus
n3: f__Neisseriaceae
n4: g__Georgfuchsia
n5: g__Sterolibacterium
n6: f__Rhodocyclaceae
n7: o__Rhodocyclales
n8: g__Sulfuricella
n9: f__Sulfuricellaceae
o0: o__Sulfuricellales
o1: c__Betaproteobacteria
o2: o__Bdellovibrionales
o3: o__Deltaproteobacteria_incertae_sedis
o4: g__Desulfatiglans
o5: g__Desulfonema
o6: f__Desulfobacteraceae
o7: g__Desulfobulbus
o8: f__Desulfobulbaceae
o9: o__Desulfobacterales
p0: g__Desulfuromonas
p1: f__Desulfuromonadaceae
p2: g__Geobacter
p3: f__Geobacteraceae
p4: o__Desulfuromonadales
p5: g__Anaeromyxobacter
p6: f__Cystobacteraceae
p7: f__Polyangiaceae
p8: o__Myxococcales
p9: g__Smithella
q0: g__Syntrophus
q1: f__Syntrophaceae
q2: g__Syntrophobacter
q3: f__Syntrophobacteraceae
q4: o__Syntrophobacterales
q5: c__Deltaproteobacteria
q6: g__Sulfuricurvum
q7: f__Helicobacteraceae
q8: o__Campylobacterales
q9: c__Epsilonproteobacteria
r0: g__Acidiferrobacter
r1: f__Ectothiorhodospiraceae
r2: o__Chromatiales
r3: g__Aquicella
r4: f__Coxiellaceae
r5: o__Legionellales
r6: g__Methylobacter
r7: g__Methylomicrobium
r8: g__Methylomonas
r9: g__Methylosarcina
s0: f__Methylococcaceae
s1: o__Methylococcales
s2: f__Moraxellaceae
s3: g__Pseudomonas
s4: f__Pseudomonadaceae
s5: o__Pseudomonadales
s6: g__Panacagrimonas
s7: g__Steroidobacter
s8: f__Sinobacteraceae
s9: g__Arenimonas
t0: g__Rudaea
t1: f__Xanthomonadaceae
t2: o__Xanthomonadales
t3: c__Gammaproteobacteria
t4: g__Spirochaeta
t5: g__Treponema
t6: f__Spirochaetaceae
t7: o__Spirochaetales
t8: c__Spirochaetia
t9: g__Opitutus
u0: f__Opitutaceae
u1: o__Opitutales
u2: c__Opitutae
u3: c__Subdivision3
u4: f__Verrucomicrobiaceae
u5: o__Verrucomicrobiales
u6: c__Verrucomicrobiae
