# Supplementary material for: Associations of soil bacterial diversity and function with plant diversity in Carex tussock wetland
Source: Front Microbiol. 2023 Mar 1;14:1142052. doi: 10.3389/fmicb.2023.1142052 (PMC10115198; doi:10.3389/fmicb.2023.1142052)
Supplement: Supplementary file 5 [file Table_1.docx]

**Supplementary Table S1** Analysis of variance of the top ten phyla.

| **Bacterial dominant phyla** | **Quadrat** | **Sample plots** | | | | | | | | |
| --- | --- | --- | --- | --- | --- | --- | --- | --- | --- | --- |
|  |  | ***NW*** | | | ***ARW*** | | | ***NRW*** | | |
| **Proteobacteria** | ***ma*** | a | 30.77±0.82 | b | c | 21.95±0.99 | b | b | 26.29±1.73 | b |
|  | ***me*** | b | 28.00±2.44 | b | b | 26.27±1.11 | a | a | 38.11±0.73 | a |
|  | ***mi*** | a | 44.65±2.49 | a | b | 23.37±1.39 | b | a | 39.22±4.36 | a |
| **Chloroflexi** | ***ma*** | c | 18.77±1.13 | a | a | 25.20±0.70 | a | b | 23.29±0.85 | a |
|  | ***me*** | b | 8.62±1.40 | c | b | 8.31±0.94 | b | a | 14.99±3.09 | b |
|  | ***mi*** | b | 16.10±1.31 | b | a | 28.07±5.94 | a | b | 21.00±0.72 | a |
| **Bacteroidetes** | ***ma*** | a | 9.36±0.76 | b | a | 11.51±2.00 | b | a | 12.18±1.66 | a |
|  | ***me*** | a | 21.08±3.35 | a | a | 24.43±1.01 | a | b | 10.73±2.57 | a |
|  | ***mi*** | a | 10.74±0.66 | b | a | 7.41±1.43 | c | a | 11.32±4.34 | a |
| **Acidobacteria** | ***ma*** | a | 8.54±0.42 | b | b | 2.99±0.07 | b | b | 3.65±0.86 | b |
|  | ***me*** | a | 15.90±2.49 | a | a | 14.71±0.64 | a | b | 11.01±0.95 | a |
|  | ***mi*** | b | 3.89±0.23 | c | a | 14.47±3.31 | a | b | 3.04±0.81 | b |
| **Planctomycetes** | ***ma*** | a | 3.56±0.36 | b | a | 4.11±0.18 | a | b | 2.39±0.45 | a |
|  | ***me*** | a | 4.22±0.29 | a | a | 4.50±0.30 | a | b | 1.70±0.17 | b |
|  | ***mi*** | a | 1.93±0.24 | c | a | 2.07±0.21 | b | b | 1.26±0.24 | b |
| **Firmicutes** | ***ma*** | c | 1.30±0.22 | b | a | 7.39±0.49 | a | b | 2.60±0.67 | a |
|  | ***me*** | b | 0.93±0.08 | b | b | 0.67±0.14 | c | a | 2.45±0.61 | a |
|  | ***mi*** | a | 4.13±0.58 | a | ab | 3.09±1.00 | b | b | 2.10±0.60 | a |
| **Actinobacteria** | ***ma*** | b | 1.32±0.32 | b | a | 2.45±0.35 | b | a | 2.48±0.30 | a |
|  | ***me*** | a | 3.41±0.37 | a | b | 2.12±0.25 | b | b | 1.95±0.43 | a |
|  | ***mi*** | b | 1.55±0.13 | b | a | 4.93±1.29 | a | b | 2.38±0.40 | a |
| **Ignavibacteriae** | ***ma*** | a | 6.61±0.39 | a | b | 3.55±0.52 | a | b | 3.32±0.49 | a |
|  | ***me*** | b | 0.55±0.22 | c | a | 1.52±0.22 | b | a | 1.53±0.51 | b |
|  | ***mi*** | a | 2.26±0.36 | b | b | 1.03±0.24 | b | b | 1.30±0.28 | b |
| **Verrucomicrobia** | ***ma*** | b | 1.47±0.09 | b | c | 0.65±0.21 | b | a | 2.36±0.31 | b |
|  | ***me*** | a | 3.27±0.62 | a | b | 1.99±0.16 | a | a | 3.15±0.31 | a |
|  | ***mi*** | b | 1.48±0.14 | b | a | 2.35±0.26 | a | a | 2.10±0.30 | b |
| **Nitrospirae** | ***ma*** | a | 0.69±0.02 | b | c | 0.03±0.01 | c | b | 0.39±0.02 | b |
|  | ***me*** | a | 1.32±0.25 | a | a | 2.06±0.25 | a | a | 1.89±0.54 | a |
|  | ***mi*** | b | 0.40±0.09 | b | a | 1.37±0.33 | b | b | 0.33±0.10 | b |

Data represent the means ± standard deviations. Analysis of variance (with Duncan’s multiple comparison test) was used to test the significance of differences. Values labeled with different lowercase letters were significant different (*p* < 0.05). The left letters correspond to comparisons of the differences among the three sample plots, and the right letters correspond to comparisons of the differences among the three quadrats within each sample plot. *NW*, natural wetland; *ARW*, artificially restored wetland; *NRW*, naturally restored wetland; *ma*, maximum plant Shannon index quadrat; *me*, median plant Shannon index quadrat; *mi*, minimum plant Shannon index quadrat. **Supplementary Table S2** βNTI scores of soil bacterial communities of *NW*, *ARW*, and *NRW* plots.

| ***NW*** | | | ***ARW*** | | | ***NRW*** | | |
| --- | --- | --- | --- | --- | --- | --- | --- | --- |
| Quadrat i | Quadrat j | βNTI score | Quadrat i | Quadrat j | βNTI score | Quadrat i | Quadrat j | βNTI score |
| *NWmi1* | *NWma1* | 1.5301 | *ARWmi3* | *ARWmi1* | 4.8071 | *NRWmi3* | *NRWmi2* | 5.1385 |
| *NWmi1* | *NWma3* | 1.4352 | *ARWmi3* | *ARWmi2* | 4.3212 | *NRWmi3* | *NRWma1* | 5.1180 |
| *NWmi1* | *NWma2* | 1.4285 | *ARWme3* | *ARWme2* | 3.4406 | *NRWmi3* | *NRWma2* | 4.9606 |
| *NWmi3* | *NWmi1* | 1.3500 | *ARWme2* | *ARWme1* | 2.7826 | *NRWmi3* | *NRWme2* | 4.4047 |
| *NWmi2* | *NWmi1* | 0.9402 | *ARWmi3* | *ARWma2* | 2.3928 | *NRWmi3* | *NRWma3* | 4.3375 |
| *NWme3* | *NWma3* | 0.6479 | *ARWma3* | *ARWma1* | 2.1680 | *NRWme2* | *NRWma1* | 4.2135 |
| *NWme3* | *NWma2* | 0.5670 | *ARWma3* | *ARWma2* | 2.0751 | *NRWme1* | *NRWma1* | 4.1640 |
| *NWme3* | *NWma1* | 0.4585 | *ARWmi3* | *ARWma1* | 1.9763 | *NRWme3* | *NRWme2* | 3.6578 |
| *NWmi3* | *NWmi2* | 0.3975 | *ARWme3* | *ARWme1* | 1.5716 | *NRWme1* | *NRWma2* | 3.4820 |
| *NWmi1* | *NWme3* | 0.1325 | *ARWme2* | *ARWma1* | 1.5517 | *NRWme2* | *NRWma2* | 3.4803 |
| *NWma2* | *NWma1* | 0.0803 | *ARWme2* | *ARWma2* | 1.4334 | *NRWmi1* | *NRWme2* | 3.3549 |
| *NWmi2* | *NWma2* | -0.1014 | *ARWme2* | *ARWma3* | 1.3811 | *NRWmi2* | *NRWme2* | 3.0535 |
| *NWmi2* | *NWme1* | -0.1881 | *ARWmi1* | *ARWme2* | 1.2785 | *NRWme3* | *NRWme1* | 2.9193 |
| *NWme3* | *NWme2* | -0.2083 | *ARWmi2* | *ARWme2* | 1.2285 | *NRWme2* | *NRWma3* | 2.2717 |
| *NWmi2* | *NWma1* | -0.2610 | *ARWmi1* | *ARWma2* | 1.2073 | *NRWme1* | *NRWma3* | 2.1492 |
| *NWmi1* | *NWme1* | -0.3705 | *ARWmi2* | *ARWma2* | 1.0690 | *NRWmi3* | *NRWmi1* | 2.0592 |
| *NWmi2* | *NWma3* | -0.4077 | *ARWmi3* | *ARWme2* | 0.7348 | *NRWmi2* | *NRWme1* | 2.0517 |
| *NWme1* | *NWma2* | -0.4212 | *ARWmi3* | *ARWma3* | 0.7259 | *NRWmi3* | *NRWme1* | 1.6029 |
| *NWmi2* | *NWme3* | -0.4964 | *ARWmi3* | *ARWme1* | 0.7021 | *NRWme3* | *NRWma1* | 1.5288 |
| *NWme2* | *NWma2* | -0.5942 | *ARWmi1* | *ARWme1* | 0.5807 | *NRWmi2* | *NRWmi1* | 1.4403 |
| *NWma3* | *NWma1* | -0.6213 | *ARWmi2* | *ARWme1* | 0.5467 | *NRWmi3* | *NRWme3* | 1.2479 |
| *NWmi3* | *NWme3* | -0.6240 | *ARWmi2* | *ARWma1* | 0.2434 | *NRWma3* | *NRWma1* | 1.0343 |
| *NWma3* | *NWma2* | -0.6243 | *ARWmi1* | *ARWma1* | 0.2322 | *NRWmi1* | *NRWme1* | 0.7786 |
| *NWmi2* | *NWme2* | -0.6489 | *ARWma2* | *ARWma1* | 0.0392 | *NRWmi1* | *NRWma1* | 0.7744 |
| *NWme2* | *NWma1* | -0.6612 | *ARWmi2* | *ARWmi1* | 0.0022 | *NRWmi2* | *NRWma1* | 0.6896 |
| *NWme1* | *NWma3* | -0.6620 | *ARWmi1* | *ARWma3* | -0.1865 | *NRWme2* | *NRWme1* | 0.4513 |
| *NWme1* | *NWma1* | -0.6660 | *ARWme1* | *ARWma1* | -0.3390 | *NRWmi1* | *NRWme3* | 0.1064 |
| *NWmi3* | *NWma2* | -0.7138 | *ARWme3* | *ARWma1* | -0.3508 | *NRWme3* | *NRWma3* | 0.0999 |
| *NWmi3* | *NWma1* | -0.7371 | *ARWmi2* | *ARWma3* | -0.4097 | *NRWma3* | *NRWma2* | 0.0738 |
| *NWmi1* | *NWme2* | -0.8015 | *ARWme3* | *ARWma2* | -0.4517 | *NRWma2* | *NRWma1* | 0.0251 |
| *NWme2* | *NWma3* | -0.8349 | *ARWme1* | *ARWma2* | -0.5192 | *NRWmi2* | *NRWma3* | -0.0001 |
| *NWme2* | *NWme1* | -0.8597 | *ARWme3* | *ARWma3* | -0.5942 | *NRWmi2* | *NRWme3* | -0.0520 |
| *NWme3* | *NWme1* | -0.8821 | *ARWmi3* | *ARWme3* | -0.5986 | *NRWmi1* | *NRWma3* | -0.2805 |
| *NWmi3* | *NWma3* | -0.9422 | *ARWmi2* | *ARWme3* | -0.6593 | *NRWmi1* | *NRWma2* | -0.2937 |
| *NWmi3* | *NWme1* | -0.9902 | *ARWmi1* | *ARWme3* | -0.7136 | *NRWmi2* | *NRWma2* | -0.5730 |
| *NWmi3* | *NWme2* | -1.2588 | *ARWme1* | *ARWma3* | -0.7820 | *NRWme3* | *NRWma2* | -0.8568 |

βNTI, β–nearest taxon index; *NW*, natural wetland; *ARW*, artificially restored wetland; *NRW*, naturally restored wetland; *ma*, maximum plant Shannon index quadrat; *me*, median plant Shannon index quadrat; *mi*, minimum plant Shannon index quadrat.
